# A mixed-integer linear programming method for time-dependent line planning in passenger railway systems

**DOI:** 10.1371/journal.pone.0322394

**Published:** 2025-05-27

**Authors:** Xin Shi, Wenliang Zhou, Xiang Li

**Affiliations:** School of Traffic and Transportation Engineering, Central South University, Changsha, Hunan, China; Beijing University of Technology, CHINA

## Abstract

This paper addresses a line planning problem (LPP) that simultaneously optimizes both train and passenger times in passenger railway systems, considering time-dependent origin-destination-period demand and passenger train choice. The problem is clearly and flexibly modeled in a physical infrastructure-based directed graph, which efficiently integrates the train operation choice and the passenger train choice. The problem is first formulated as a mixed-integer, non-concave, and non-linear programming model aimed at minimizing both the total operating cost of trains and the total travel cost of passengers. To solve the problem, an extended time-dimension method is proposed to transform the non-concave and non-linear model into a mixed-integer linear programming (MILP) model that can be solved using a commercial solver. Additionally, a set of simplification strategies is introduced to reduce the computational complexity while ensuring the global optimality of the linear model. A case study of a busy Chinese railway line demonstrates that the optimized time-dependent line plan enhances operational efficiency and accommodates the diversified travel preferences driven by time-dependent demand.

## 1 Introduction

Railway transportation is a major and essential mode of transport in many countries. For instance, in 2024, China’s railway passenger turnover reached 15,799.1 billion passenger-kilometers, and the operating mileage of high-speed railway (HSR) reached 48,000 kilometers. This highlights the irreplaceable role of the passenger railway system in driving economic and social development [1–2]. Li et al. [3] categorize the railway system planning problem into various stages, such as network design, line planning, train scheduling. The line planning problem (LPP) is a crucial and challenging optimization problem in the passenger railway transport due to its large scale, practical significance, and its close connection with passenger train choice (PTC).

The LPP typically focuses on selecting a set of train lines, along with the operation frequencies and stop schedules for those lines within a passenger railway system. In essence, a train line refers to a specific route serviced by trains, specifying the stations visited by the trains. The operation frequency of a train line determines the number of practical trains running along the route within a given time range, while the stop schedule dictates the stations where trains stop and the sequence of these stops. Different trains operating along the same route may follow distinct stop plans. A well-structured line plan dictates the arrangement of trains and their respective numbers on the railway system, influencing both the operational costs of the trains and the travel costs for passengers. Therefore, achieving a balanced line plan with multiple decision-making objectives is crucial for providing high-quality services to both railway operators and passengers.

From an optimization perspective, the LPP can be broadly divided into two categories: operator-oriented optimization and passenger-oriented optimization [4–5]. Research in these two areas has developed extensively. Several studies have reviewed the models and methods used in these areas [6–8]. Operator-oriented models primarily aim to minimize train operating costs while meeting transportation capacity and service requirements. Claessens et al. [9] presented an integer linear model for minimizing the operating costs, solving it using a branch and bound procedure. Goossens et al. [10] developed multi-type integer programming models for operating costs minimization and solved them using CPLEX on three real-life instances. Torres et al. [11] introduced a flow-based model to minimize the operating costs, which was solved with IP-solver SCIP. Canca et al. [12] proposed a model to minimize speed-dependent variable operating costs, solved using an iterative sequential branch-and-cut method.

Passenger-oriented models focus on improving passenger service levels, typically under constraints on train capacity. These models may involve maximizing direct passenger routes or minimizing travel and transfer times. Bussieck et al. [13] proposed a MILP model to maximize the number of direct travelers and solved it using the CPLEX solver. Schöbel and Scholl [14] introduced an integer model and applied a Dantzig‒Wolfe decomposition method. Schmidt and Schöbel [15] integrated LPP with PTC to jointly minimize passenger travel time. Szeto and Wu [16], along with Szeto and Jiang [17] designed mixed-integer and bi-level programming models, respectively, solving them with genetic and hybrid artificial bee colony algorithms. Xie et al. [18] proposed a non-linear model for a new joint problem aimed at minimizing passenger costs under train operating cost constraints, and solved using an iterative heuristic.

In recent years, some studies have attempted to combine operator-oriented and passenger-oriented goals. Guan et al. [19] developed a linear integer programming model for optimizing the line route and passenger route assignment, integrating both operator-oriented goal and the passenger-oriented goal. Borndörfer et al. [20] constructed a multicommodity flow model for optimizing line routes and frequencies with passengers’ free path choices, solved using a column-generation algorithm. Gallo et al. [21] proposed four non-linear models for optimizing line frequencies and the stop schedules under elastic demand, solving them with two different algorithms. Canca et al. [22] introduced a mixed-integer non-linear programming model for optimizing line frequencies and the stop schedules, considering passenger route choices, solved using an extended cutting plane algorithm. Goerigk and Schmidt [23] developed a MILP model to optimize train routes, frequencies, and stop schedules with passenger route choices, solving it with an ε-constraint method and genetic algorithm. Zhou et al. [5] presented a MILP model for optimizing train routes and frequencies with passenger path assignment, solved using an outer approximation method.

In recent years, both train operators and passengers have increasingly focused on train/passenger time during railway operations, particularly in HSR systems. Operators emphasize the accuracy of train arrival/departure times when designing line plans, as this can significantly benefit train timetabling in subsequent stages. If train arrival/departure times are integrated into the LPP, it will allow for more targeted timetabling, while avoiding the large solution space that results from joint optimization of LPP and timetabling. On the other hand, passengers are concerned with their preferred departure times, in-vehicle time, and waiting time. If passenger time preferences are integrated into line planning, PTC can be more accurately delineated and incorporated into the optimization process. Considering both train and passenger time will allow for a more seamless integration of train operation choices (TOC) and PTC, leading to better line plans that balance multi-objective goals.

However, few studies have taken both train and passenger time into account within the LPP. Su et al. [24] and Zhao et al. [25] developed bi-level models for the LPP, incorporating time-dependent demand, and solved the non-concave and non-linear models using simulated annealing algorithm. Nevertheless, three critical issues remain unaddressed in these studies. First, heuristic algorithms often struggle to predict the deviation between the obtained solution and the global optimal solution, especially for large-scale practical problems where finding the global optimal solution within a reasonable timeframe is difficult. Second, passenger flow allocation does not ensure that all passengers under time-dependent demand can complete their journeys. Third, the fixed costs associated with train operations are not considered in the objective function.

Table 1 gives the main decisions involved in this study and highlights its contributions to existing literature. This paper integrates train and passenger times in the LPP, aiming to reconcile operator-oriented and passenger-oriented goals with a universal passenger railway system. The problem is formulated as a MILP method for the first time to achieve a global optimal solution, fully combining TOC and PTC under time-dependent demand. The primary contributions are as follows:

**Table 1:** Table of main elements of different papers.

Paper	Transit mode	Demand model	Decisions				Operator-oriented		Passenger-oriented			Model	Solution method	Global optimization algorithm
			Line route	Line frequency	stop schedule	Train/passenger time	Fixed cost	Frequency related cost	In-vehicle time	Transfer time	Waiting time			
[19]	Urban rail	Daily demand	Y	N	N	N	Y	N	Y	Y	N	0–1 integer linear programming	CPLEX	Y
[20]	Bus and tram	Daily demand	Y	Y	N	N	Y	Y	Y	N	N	MILP	Column-generation algorithm	Y
[21]	Mixed-transit	Elastic demand	N	Y	Y	N	N	Y	Y	N	Y	0–1 integer non-linear programming	Meta-heuristic method	N
[22]	Railway	Daily demand	N	Y	Y	N	Y	Y	Y	Y	Y	MINLP	Extended cutting plane	N
[23]	Railway	Daily demand	Y	Y	Y	N	Y	Y	Y	Y	Y	Bi-level programming and MILP	ε-constraint method + CPLEX and genetic algorithm	Y
[24]	Railway	Time-dependent demand	Y	Y	Y	Y	N	Y	Y	N	Y	Bi-level programming	Simulated annealing algorithm	N
[5]	Urban rail	Daily demand	Y	Y	N	N	Y	Y	Y	Y	Y	MILP	Outer approximation method + CPLEX	Y
[25]	Railway	Time-dependent demand	Y	Y	Y	Y	N	Y	Y	Y	Y	Bi-level programming	Simulated annealing algorithm	N
This paper	Railway	Time-dependent demand	Y	Y	Y	Y	Y	Y	Y	Y	Y	MILP	Extended time-dimension method + GUROBI	Y

(1) This study establishes a rigorous mathematical model for the LPP that simultaneously optimizes train/passenger time in a universal passenger railway system with PTC under time-dependent origin-destination-period demand. The model is described in a physical infrastructure-based directed graph, which integrates TOC considering train arrival/departure times and PTC considering passenger time preferences. This approach not only enhances operational efficiency for operators but also accommodates the diversified travel preferences of passengers.
(2) This is the first study to propose a MILP method to solve the time-dependent demand-driven line planning problem, considering diverse goals and achieving a global optimal solution in a passenger railway system. A set of simplification strategies is introduced to improve MILP efficiency by expanding a few arcs in the directed graph. A case study of a busy HSR line in China illustrates the effectiveness of this method.

Here, Y stands for Yes, that is, the study considered the element, and N stands for No, that is, the study did not consider the element.

The remaining section of this paper is structured in the following. Section 2 describes the LPP with train/passenger time under time-dependent demand in a directed graph. Section 3 formulates the mixed-integer non-concave and non-linear programming model. Section 4 presents an extended time-dimension method to convert the non-concave and non-linear model into a MILP model and introduces simplification strategies to reduce computational complexity. Section 5 provides a case study of a busy HSR line in China to demonstrate the MILP method. Section 6 concludes the paper and outlines future research directions.

## 2 Problem statement

In this section, we construct a directed graph based on the physical infrastructure of the railway system and candidate trains, which effectively models the integration of the TOC and PTC. Table 2 lists the main notations used throughout the paper.

**Table 2:** Notation for the railway system, candidate trains, passenger travel network, and time-dependent demand.

Object	Symbol	Description
Railway system	S	Set of stations in the railway system
	$k,k^{\prime },r,s$	Index of station, where $k,k^{\prime },r,s\in S$
	E	Set of sections in the railway system
	$\left(k,k^{\prime }\right)$	Index of a section from two neighboring stations k to $k^{\prime }$, where $(k,k^{\prime })\in E$
Candidate train	F	Set of candidate trains
	f	Index of a candidate train, where f∈F
	$o_{f}$, $d_{f}$	Origin and destination stations of candidate train f
	$S_{f}$	Set of stations visited by candidate train f∈F
	$E_{f}$	Set of sections visited by candidate train f∈F
	${Cap}_{f}$	Passenger service capacity of candidate train f∈F
	$t_{f}^{s}$, $t_{f}^{e}$	Earliest and latest departure times of candidate train f∈F from the origin station
	$\tau_{f}^{k,k^{\prime }}$	Running time of candidate train f∈F on section $\left(k,k^{\prime }\right)\in E_{f}$ without considering acceleration and deceleration
	$w_{f}^{k}$	Dwell time of candidate train f∈F at station $k\in S_{f}$, including both deceleration and acceleration times
Passenger travel network	N	Set of nodes, any type of node i,j∈N
	$N_{S}$	Set of station nodes, where $N_{S}\in N$
	$n_{k}$	Index of the station node corresponding to station k
	$u_{f}^{k}$	Index of the arrival node corresponding to the arrival of candidate train f∈F at station $k\in S_{f}$
	$v_{f}^{k}$	Index of the departure node corresponding to the departure of candidate train f∈F at station $k\in S_{f}$
	A	Set of arcs
	$\left(n_{k},v_{f}^{k}\right)$	Boarding arc for passengers getting on train f at station k
	$\left(v_{f}^{k},u_{f}^{k^{\prime }}\right)$	Travel arc for passengers traveling from station k to station $k^{\prime }$ by train f
	$\left(u_{f}^{k},v_{f}^{k}\right)$	Dwell arc for passengers stopping at station k with train f
	$\left(u_{f}^{k},v_{f^{\prime }}^{k}\right)$	Transfer arc for passengers transferring from train f to another train $f^{\prime }$ at station k
	$\left(u_{f}^{k},n_{k}\right)$	Getting-off arc for passengers getting off train f at station k
	${\hat{A}}_{i}$	Set of arcs departing from node i in the directed graph
	${\overset{ˇ}{A}}_{i}$	Set of arcs entering node i in the directed graph
Time-dependent demands	$H_{k}$	Set of time periods during which passengers arrive at origin station k
	h	Index of time period, where $h\in H_{k}$
	${ℛ}_{h}$	Midpoint of time period h
	$q_{r,s}^{h}$	Number of passengers traveling from stationrto stations, entering station r in time period h∈H

Some assumptions are given as follows.

(A1) The time-dependent demand of each OD pair in each time period is fixed, and passengers choose trains based on the minimum generalized travel cost. This is a common assumption in both LPP and timetabling problem [5,14,26]. However, the actual number of passengers on each train is influenced by the PTC.

(A2) An optimal line plan can be chosen from a set of pre-determined candidate trains that satisfy the transportation capacity constraints of the railway section. This assumption is widely used in existing literature [5,20,27].

(A3) Passengers in each time period are assumed to arrive at their origin station at the same time (with the expected departure time being the midpoint of each time period). In other words, passengers may depart after the midpoint when boarding their respective trains. The dwell time of each train at each station is fixed, as is commonly assumed in similar studies [25].

### 2.1 Railway system and candidate train

The physical railway system under consideration is represented by a set of stations S and sections E. Each station k∈S is assumed to have sufficient capacity to accommodate both stopping and passing trains, and each section $\left(k,k^{\prime }\right)\in E$ is assumed to have two one-way tracks.

A set of candidate trains, denoted as F, has been pre-determined based on the space‒time distribution of passengers within the physical railway system. The relevant notations for candidate train, including the origin, destination, passenger service capacity, and sequence of visited stations, is provided in Table 2. The symbols $t_{f}^{s}$ and $t_{f}^{e}$ represent the earliest and latest departure times of train f from its origin station $o_{f}$. The travel time $\tau_{f}^{k,k^{\prime }}$ of each candidate train f in section $\left(k,k^{\prime }\right)$ only considers the running time and puts the additional time together into the dwell time $w_{f}^{k}$.

We define $\tau_{f}^{k,k^{\prime }}$ as the pure time, and $w_{f}^{k}$ as the time consumed by train f stopping at station k, which consists of three parts: the required dwell time at station k, the additional time for decelerating to stop at station k, and the additional time for accelerating from station k. Obviously, if a station is not a stop station, then the time consumed there is 0. Note that the acquisition of the train running time in sections and the time consumed at stations in this paper is only for calculating the train travel time between each two visited stations, instead of generating a workable train timetabling. Obviously, this simplification does not affect the calculation of the train travel time.

The decisions that need to be made for each candidate train in this study include the following three aspects:

(1) Whether is the train is selected to operate.
(2) Whether the train stops at each of its visiting stations when it is selected to operate.
(3) The departure and arrival times at each visited station when the train is selected to operate.

### 2.2 Passenger travel network

The passenger travel network is introduced to model the PTC and travel costs, and it represented by a directed graph. This directed graph consists of three types of nodes and five types of directed arcs, denoted as N and A, respectively. Given a physical railway system and a set of given candidate trains, the directed graph is defined as follows.

#### (1) Nodes.

The first type of nodes, named as station nodes, are constructed for stations, and the two other types of nodes, called the departure node and arrival node respectively, are generated for the departure and arrival operations of candidate trains at their visiting stations. Denote $N_{S}$ as a set of station nodes, and $n_{k}\in N_{S}$ as a station node corresponding to station k. Moreover, denote $v_{f}^{k}$ and $u_{f}^{k}$ as the departure and arrival node of train f at station $k\in S_{f}$. At the same time, we define the arrival and departure times of train f at station $k\in S_{f}$ in the space‒time network as $a_{f}(k)$ and $d_{f}(k)$ respectively. For convenience, both symbols i and j are defined to represent a node of any type.

#### (2) Directed arcs.

Boarding arc: Each boarding arc $\left(n_{k},v_{f}^{k}\right)$ is constructed from a station node to a departure node corresponding to the same station, representing the process of passengers boarding a train. The capacity of each boarding arc $\left(n_{k},v_{f}^{k}\right)$ is related to whether the train f is selected to run and whether the train f stop at the station k. If both yes, then its capacity is set as the ${Cap}_{f}$; otherwise, it is 0, which means no passenger is able to visit it.

Travel arc: Each travel arc $\left(v_{f}^{k},u_{f}^{k^{\prime }}\right)$ is constructed from a departure node to an arrival node at its two neighboring visiting stations of the same candidate train, it shows the process of passengers taking a train from one stop to the next stop. The capacity of each travel arc $\left(v_{f}^{k},u_{f}^{k^{\prime }}\right)$ is related to whether the train f is selected to run. If yes, then its capacity is set as the ${Cap}_{f}$; otherwise, it is 0.

Dwell arc: Each dwell arc $\left(u_{f}^{k},v_{f}^{k}\right)$ is constructed from an arrival node to a departure node at the same station of the same candidate train, and it shows the process of passengers staying at the stopping station. The capacity of each dwell arc $\left(u_{f}^{k},v_{f}^{k}\right)$ is related to whether the train f is selected to run. If yes, its capacity is set as the ${Cap}_{f}$; otherwise, it is 0.

Transfer arc: Each transfer arc $\left(u_{f}^{k},v_{f^{\prime }}^{k}\right)$ is constructed from an arrival node of a candidate train to a departure node of another candidate train at the same station, and it shows the process of passengers changing from one train to another train at a transfer station. For the transfer arc $\left(u_{f}^{k},v_{f^{\prime }}^{k}\right)$, its capacity is related to: (i) whether both trains are selected to run and whether both stop at the station; (ii) whether the arrival time and departure time of the two candidate trains at stationksatisfy the requirement of the minimum transfer time. If both yes, then its capacity is set as the ${Cap}_{f^{\prime }}$; otherwise, it is 0.

Getting-off arc: Each getting-off arc $\left(u_{f}^{k},n_{k}\right)$ is constructed from an arrival node to its corresponding station node, and it shows the process of passengers getting off a candidate train. The capacity of $\left(u_{f}^{k},n_{k}\right)$ is related to whether the train f is selected to run and whether the train f stop at the station. If both yes, then its capacity is set as the ${Cap}_{f}$; otherwise, it is 0.

For convenience of description, we denote ${\hat{A}}_{i}$ and ${\overset{ˇ}{A}}_{i}$ as the sets of directed arcs departing from and entering into node i respectively on the directed graph, and (i,jas an arc of any type on the directed graph.

**Fig 1:**
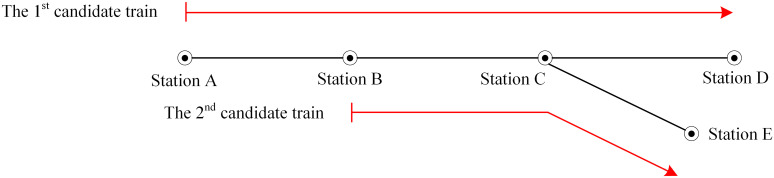
A railway system and two candidate trains.

**Fig 2:**
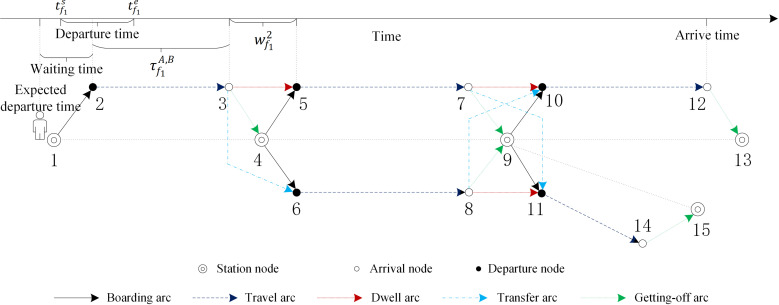
The directed graph corresponding toFig 1.

To further clarify the directed graph, Fig 1 gives a Y-shaped physical railway system consisting of five train stations and four sections, and two candidate trains. Fig 2 presents a directed graph corresponding to Fig 1. The station nodes 1, 4, 9, 13, 15 correspond to stations A, B, C, D, E respectively, and departure nodes 2, 5, 10 and arrival nodes 3, 7, 12 respectively of the 1^st^ candidate train at its visited stations, while nodes 6, 11 and 8, 14 are these of the 2^nd^ candidate train. Regarding the directed arcs of the 1^st^ candidate train, they consist of boarding arcs (1,2), (4,5), (9,10)(1,2,4,5,9,10); getting-off arcs (3,4), (7,9), (12,13)(3,4,7,9,12,13); travel arcs (2,3), (5,7), (10,12)(2,3,5,7,10,12); and dwell arcs (3,5), (7,10)(3,5,7,10). The directed arcs of the 2^nd^ candidate train are composed of boarding arcs (4,6), (9,11)(4,6,9,11); travel arcs (6,8), (11,14)(6,8,11,14); dwell arcs (8,118,11); and getting-off arcs (8,9), (14,15)(8,9,14,15). In addition, the transfer arcs (3,63,6) and (7,117,11) are from the 1^st^ candidate train to the 2^nd^ candidate train at station B and C respectively, while transfer arc (8,108,10) is from the 2^nd^ candidate train to the 1^st^ candidate train at station C. Obviously, at most one of the transfer arcs (7,117,11) and (8,108,10) is valid because of the required minimum transfer time for passengers.

Note that the construction of the three nodes and five arcs is related to the physical railway system and candidate trains, but irrelevant to the TOC of candidate trains. Thus, the scale of the passenger travel network depends on both the computation scales of the railway system and candidate trains in the input data. However, the availability of arcs and passengers’ travel costs such as travel time on arcs rest with the TOC of candidate trains.

### 2.3 Time-dependent demand and passenger train choice

Passengers arrive at their origin stations at different times throughout the day, which means they have different expected departure times. These times are divided into multiple time periods. Many studies [25,27] divide the different times into multiple time periods by a fixed scale (e.g., one hour). Denote $H_{r}$ as a set of time periods that represents the time range at which a passenger enters its origin station r. Then, according to the set of time periods, passengers departing from station r to station s in the fixed time range can be divided into lots of time-dependent demands. Denote $q_{r,s}^{h}$ as the number of passengers departing from the station r to the station s in the time period $h\in H_{r}$. The expected departure time for passengers in the same time period $h\in H_{r}$ entering the origin station r is uniformly set as the middle time ${ℛ}_{h}$ of a time period h, although the entering time is different. The time dimension used in the time period h and the time in the space‒time network are both measured in minutes. h represents the expected boarding time of each passenger flow, while $a_{f}(k)$ and $d_{f}(k)$ represent the arrival and departure times of the train at a specific station in the space‒time network. Note that passengers departing from different origin stations can have different sets of entering time periods, but in this paper all the origin stations have the same set of time periods.

The costs faced by passengers when traveling from their origins to destinations during a specific time period depend on the arcs they traverse within the passenger travel network. Passengers need to consider: (i) the waiting time on boarding arcs, (ii) travel time and ticket price on travel arcs (which depend on the train running time of the section and fare rate), (iii) dwell time on dwell arcs, and (iv) transfer time on transfer arcs, which depends on transfer time. In addition, passengers do not have costs on a getting-off arc.

**Fig 3:**
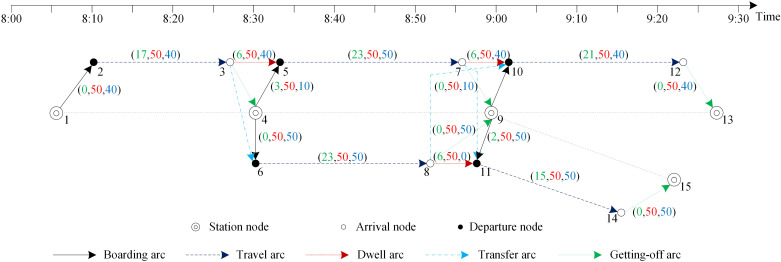
A case illustrating the PTC.

For the time-dependent demand of each time period between two stations, passengers usually choose a route with the lowest total travel costs to travel at the limit of the arcs’ capacities. There are 150 passengers in Fig 3, and the demand is given in Table 3. The departure time range of the two candidate trains is given as 8:00‒9:00. The green, red, and blue numbers in brackets in Fig 3 represent the passenger travel cost, passenger service capacity, and the number of passengers of the corresponding arcs (note that the information of the arcs that are not chosen is not given). For 60 passengers from station B to station C, there are two routes to complete the trip, called (4,64,6) → (6,86,8) → (8,9) and (4,5) → (5,75,7) → (7,97,9), but because of the capacity constraints on the arc, 10 of them need to choose the second most expensive route.

**Table 3:** Time-dependent demand and PTC corresponding to Fig 3.

Time-dependent demand				Passenger train choice		
Origin station	Destination station	Expected departure time	Quantity	Train choice	Route choice	Cost
A	D	8:10	40	1^st^ train	(1,2) → (2,3) → (3,53,5) → (5,75,7) → (7,107,10) → (10,1210,12) → (12,13)	73
B	C	8:30	60	1^st^ train and 2^nd^ train	(4,64,6) → (6,86,8) → (8,9) or (4,5) → (5,75,7) → (7,97,9)	23 or 26
C	E	8:55	50	2^nd^ train	(9,119,11) → (11,1411,14) → (14,15)	17

Therefore, for each time-dependent demand between two stations in any time period, we need to search its PTC combined with the TOC of candidate trains, in other words, need to decide the number of passengers visiting each arc, to make their total travel costs as low as possible.

## 3 The optimization model

In this section, a primordial mixed-integer non-concave and non-linear programming model is established for the LPP, which simultaneously optimizes both the train and passenger times in a universal passenger railway system, subject to several constraints.

### 3.1 Decision variables

The decision variables for the candidate train and time-dependent demand have been detailed in Section 2.1 and Section 2.3. These are summarized as follows:

(1) The TOC for each candidate train includes whether it is selected to run; if yes, then further decide whether it stops at each visiting station, and its arrival and departure times there.
(2) The PTC for each time-dependent demand is the number of passengers visiting each arc on a directed graph.
(3) For deciding the validity of the transfer arc, an intermediate 0–1 decision variable is used for describing whether a transfer arc is valid. Meanwhile, another intermediate 0–1 decision variable is defined for describing whether passengers are allowed to enter a boarding arc at a station in a specified time period.

Table 4 gives the detailed definitions for all the required decision variables.

**Table 4:** Definitions of decision variables.

Objects	Symbols	Definitions
Candidate train	$x_{f}$	0-1 variable, if the train is selected to run, $x_{f}=1$; otherwise, it is 0
	$y_{f}(k)$	0-1 variable, if the train is selected to run and stop at the station, then $y_{f}(k)=1$; otherwise, it is 0
	$a_{f}(k)$	Arrival time of train f∈F at the station $k\in S_{f}$.
	$d_{f}(k)$	Departure time of train f∈F at the station $k\in S_{f}$.
Passenger	$\rho_{r,s}^{h}(i,j)$	The number of passengers visiting arc (i,j) from stationrto stations in time period $h\in H_{r}$
Travel network	z(i,j)	0-1 variable, if a transfer arc $(i,j)\in A_{H}$ is valid when it satisfies some constraints detailed in the later, then z(i,j)=1; otherwise, it is 0
	$w_{h}(i,j)$	0-1 variable, if a boarding arc (i,j) allows passenger entering into an origin station in time period h, then $w_{h}(i,j)=1$, otherwise, it is 0

### 3.2 Operator-and-passenger-oriented objective

The objective function consists of two costs: the total operating cost of trains H and the total travel cost of passengers $\begin{array}{c} 𝛹 \end{array}$. H consists of the organization cost (fixed cost), which is not related to the train head time; and the train travel cost (variable cost), which is related to the train head time. The train head time of a train is the time between the departure time of the train at its origin and the arrival time of the train at its destination. $\begin{array}{c} 𝛹 \end{array}$ includes the in-vehicle time, waiting time at the origin station, transfer time, and the equivalent time cost of the ticket price. The objective function of minimization is to minimize a weighted sum of the total train operational cost and the total passenger travel cost:


$$\begin{array}{c} \min G=\omega \times H+(1-\omega )\times 𝛹 \end{array}$$ (1)

where ω is a weight coefficient whose value is located in [0, 1]. When it is 0, the objective does not consider the total operating cost of trains, so it is a passenger-oriented model; and when it is 1, the objective does not consider the cost of passengers, so it is an operator-oriented model.

Let us define $\phi_{f}$ as the organization cost and $\pi_{f}$ as the train travel cost per unit of head time, and the H can be expressed as follows:


$$H=\sum_{f}\left\{x_{f}\times \phi_{f}+\pi_{f}\times \left(\sum_{\left(k,k^{\prime }\right)\in E_{f}}x_{f}\times \tau_{f}^{k,k^{\prime }}+\sum_{k\in S_{f}}y_{f}(k)\times w_{f}^{k}\right)\right\}$$ (2)

If the train f is not selected to run, namely $x_{f}=0$ and $y_{f}(k)=0$ for any visiting station k, then the train has no total operating cost.

The total travel cost of passengers is the sum of the time cost and equivalent time cost of the ticket price. Thus, it can be expressed as follows:


$$\Psi =\sum_{f}\sum_{r}\sum_{s\in S_{f}\backslash \{{\text{o}}_{\text{f}}\}}\sum_{h\in H_{r}}\rho_{r,s}^{h}\left(u_{f}^{k},n_{k}\right)\times \left(a_{f}(k)-R_{h}\right)$$

$$+\sum_{r}\sum_{s\neq r}\sum_{h\in H_{r}}\sum_{\left(v_{f}^{k},u_{f}^{k^{\prime }}\right)\in A_{T}}\rho_{r,s}^{h}\left(v_{f}^{k},u_{f}^{k^{\prime }}\right)\times (l_{k,k^{\prime }}\times \mu_{f})/\sigma$$ (3)

where $l_{k,k^{\prime }}$ is the mileage from station k to $k^{\prime }$, $\mu_{f}$ is the price rate per mileage of train f, and σ is the average time value of passengers; for its value, refer to [28].

Specifically, the first part of Eq (3) is the passenger travel time, which can be obtained by calculating the difference between the passenger’s arrival time at destination stations and the boarding time at origin stations in all getting-off arcs $\left(u_{f}^{k},n_{k}\right)$. The second part is to convert the ticket cost of all passengers reflected in all travel arcs $\left(v_{f}^{k},u_{f}^{k^{\prime }}\right)$ into time cost.

### 3.3 Constraints

Two types of constraints are considered in this model. One is established for the TOC, consisting of the time constraint of a train departing from the origin station, constraints of train arrival/departure time, and consistency constraints between the TOC and stop choice. The other is established for the PTC, consisting of flow balance constraints, constraints of passengers visiting boarding arcs, and constraints of the passenger service capacity on arcs.

#### (1) Group I: Time constraint of train departing from origin station.


$$t_{f}^{s}\times x_{f}\leq d_{f}\left(o_{f}\right)\leq t_{f}^{e}\times x_{f},\forall f\in F$$ (4)

Constraint (4) ensures that each train f is selected to operate from its origin station $o_{f}$ between the earliest and the latest departure time. However, if it has not been selected to operate, then its departure time is set to 0.

#### (2) Group II: Constraints of train arrival/departure time.


$$a_{f}\left(k^{\prime }\right)-d_{f}(k)=\tau_{f}^{k,k^{\prime }}\times x_{f},\forall f\in F;\forall (k,k^{\prime })\in E_{f}$$ (5)

$$d_{f}(k)-a_{f}(k)=w_{f}^{k}\times y_{f}(k),\forall f\in F;\forall k\in S_{f}\backslash \{o_{f},d_{f}\}$$ (6)

If train f is selected to run, constraint (5) ensures that the time range from its departure time at station k to its arrival time at the next station $k^{\prime }$ is equal to the running time $\tau_{f}^{k,k^{\prime }}$ in section $\left(k,k^{\prime }\right)\in E_{f}$; otherwise, it should be 0. And constraint (6) ensures that the time range from its arrival time $a_{f}(k)$ at station k to its departure time $d_{f}(k)$ at station k is just the consumed time $w_{f}^{k}$ if it stops at station k, namely $y_{f}(k)=1$; otherwise, it should be 0.

#### (3) Group III: Consistency constraints between train operation choice and stop choice.


$$\sum_{k\in S_{f}}y_{f}(k)\leq x_{f}\times {ℒ}_{f},\forall f\in F$$ (7)

$$y_{f}\left(o_{f}\right)+y_{f}\left(d_{f}\right)=x_{f}\times 2,\forall f\in F$$ (8)

Constraint (7) ensures that if train f∈F is not selected to run, namely $x_{f}=0$, then all its stop choices must as not, that is, $\sum k\in S_{f}y_{f}(k)=0$; otherwise, it must stop at the origin and destination stations, which is ensured by constraint (8) and it can either stop or not at other visiting stations.

#### (4) Group IV: Flow balance constraints.


$$\sum_{\left(n_{r},j\right)\in {\hat{A}}_{n_{r}}}\rho_{r,s}^{h}\left(n_{r},j\right)=q_{r,s}^{h},\forall r\neq s\in S;\forall h\in H_{r}$$ (9)

$$\sum_{(i,j)\in {\overset{ˇ}{A}}_{j}}\rho_{r,s}^{h}(i,j)=\sum_{\left(j,i^{\prime }\right)\in {\hat{A}}_{j}}\rho_{r,s}^{h}\left(j,i^{\prime }\right),\forall r\neq s\in S;\forall h\in H_{r};j\in N\backslash \left\{n_{k}:k\in S\right\}$$ (10)

$$\sum_{\left(i,n_{s}\right)\in {\overset{ˇ}{A}}_{n_{s}}}\rho_{r,s}^{h}\left(i,n_{s}\right)=q_{r,s}^{h},\forall r\neq s\in S;\forall h\in H_{r}$$ (11)

For passengers from station r to station s in time period $h\in H_{r}$, constraint (9) ensures that they have to depart from the station node $n_{r}$ with boarding arcs, constraint (11) ensures that they finally arrive at the station node $n_{s}$ with the connected getting-off arcs, and constraint (10) ensures the number of them entering into an arrival node or a departure node should be equal to the number leaving from there.

#### (5) Group V: Constraints of passengers visiting boarding arcs.


$$0\leq \sum s\rho_{k,s}^{h}\left(n_{k},v_{f}^{k}\right)\leq w_{h}\left(n_{k},v_{f}^{k}\right)\times {Cap}_{f},\forall f\in F;\forall k\in S_{f}\backslash \{d_{f}\};\forall h\in H_{k}$$ (12)

Constraint (12) ensures that only if a boarding arc $\left(n_{k},v_{f}^{k}\right)$ allows passengers to enter origin station k in time period h, namely, $w_{h}\left(n_{k},v_{f}^{k}\right)=1$, then the number of passengers visiting it can be more than 0; otherwise, it must be 0.

For the boarding arc $\left(n_{k},v_{f}^{k}\right)$, only when train f departs from station k after the middle time ${ℛ}_{h}$ of time period h, it is available for passengers entering into station k in time period h. In other words, if $w_{h}\left(n_{k},v_{f}^{k}\right)=1$, then the departure time $d_{f}(k)$ of train f at station k must be not less than ${ℛ}_{h}$. Thus, the following constraint (13) must be satisfied.


$$d_{f}(k)\geq w_{h}\left(n_{k},v_{f}^{k}\right)\times {ℛ}_{h},\forall f\in F;\forall k\in S_{f}\backslash \{d_{f}\};\forall h\in H_{k}$$ (13)

#### (6) Group VI: Constraints of passenger service capacity on various types of arcs.

(i ) Capacity constraint on boarding arcs

$$\sum_{s}\sum_{h\in H_{k}}\rho_{k,s}^{h}\left(n_{k},v_{f}^{k}\right)\leq y_{f}(k)\times {Cap}_{f},\forall f\in F;\forall k\in S_{f}\backslash \{d_{f}\}$$ (14)

Constraint (14) ensures that passengers can get on train f at station k only when this train stops there, namely, $y_{f}(k)=1$; otherwise, if this train does not stop there, no passengers can board the train through the boarding arc. Specifically, the number of passengers visiting boarding arc $\left(n_{k},v_{f}^{k}\right)$ must be 0 when $y_{f}(k)=0$, and it can be either 0 or more than 0 when $y_{f}(k)=1$.

(ii) Capacity constraints on travel arcs and dwell arcs

$$\sum_{r,s}\sum_{h\in H_{r}}\rho_{r,s}^{h}\left(v_{f}^{k}{,u}_{f}^{k^{\prime }}\right)\leq x_{f}\times {Cap}_{f},\forall f\in F;\forall \left(k,k^{\prime }\right)\in E_{f}$$ (15)

$$\sum r,s\sum h\in H_{r}\rho_{r,s}^{h}\left({u_{f}^{k},v}_{f}^{k}\right)\leq x_{f}\times {Cap}_{f},\forall f\in F;\forall k\in S_{f}\backslash \left\{o_{f},d_{f}\right\}$$ (16)

Constraint (15) ensures that if train f∈F is selected to operate, namely, $x_{f}=1$, then the passenger service capacity of travel arc $\left(v_{f}^{k}{,u}_{f}^{k^{\prime }}\right)$ is ${Cap}_{f}$, and the number of passengers visiting this arc, namely traveling by train f in section $\left(k,k^{\prime }\right),$ cannot exceed ${Cap}_{f}$; otherwise, if the train does not operate, no passengers are allowed to visit the travel arc. In the same way, constraint (16) is similar to constraint (15).

(iii) Capacity constraints on transfer arcs

$$\begin{array}{c} \sum_{r,s}\sum_{h\in H_{r}}\rho_{r,s}^{h}\left({u_{f}^{k},v}_{f^{\prime }}^{k}\right)\leq z\left({u_{f}^{k},v}_{f^{\prime }}^{k}\right)\times {Cap}_{f},\forall f^{\prime }\neq f\in F;\forall k\in S_{f}\cap S_{f^{\prime }} \end{array}$$ (17)

Constraint (17) ensures that when transfer arc $\left({u_{f}^{k},v}_{f^{\prime }}^{k}\right)$ is valid, namely, $z\left({u_{f}^{k},v}_{f^{\prime }}^{k}\right)=1$, then the number of passengers on it can be more than 0; otherwise, no passengers can visit it.

For transfer arc $\left({u_{f}^{k},v}_{f^{\prime }}^{k}\right)$, the valid variable $z\left({u_{f}^{k},v}_{f^{\prime }}^{k}\right)$ is defined to show whether it is valid or not. It is valid only when it satisfies the following two constraints.

(a) Both trains f and $f^{\prime }$ stop at station k:


$$\begin{array}{c} z\left({u_{f}^{k},v}_{f^{\prime }}^{k}\right)\leq \frac{\left[y_{f}(k)+y_{f^{\prime }}(k)\right]}{2},\forall f^{\prime }\neq f\in F;\forall k\in S_{f}\cap S_{f^{\prime }} \end{array}$$ (18)

(b) The time range from the arrival of train f to the departure of train $f^{\prime }$ at station k is more than the minimum required time for passengers transferring at this station, that is:


$$d_{f^{\prime }}(k)-a_{f}(k)\geq \Upsilon_{k}-\left[1-z\left({u_{f}^{k},v}_{f^{\prime }}^{k}\right)\right]\times M,\forall f^{\prime }\neq f\in F;\forall k\in S_{f}\cap S_{f^{\prime }}$$ (19)

Specifically, constraint (18) ensures that when both trains f and $f^{\prime }$ stop at station k, namely, $y_{f}(k)=1$ and $y_{f^{\prime }}(k)=1$, then $z\left({u_{f}^{k},v}_{f^{\prime }}^{k}\right)$ can be either 0 or 1, depending on constraint (19); otherwise, if either of them does not stop there, then it can only be 0. Constraint (19) ensures that the time range from departure time $d_{f^{\prime }}(k)$ to arrival time $a_{f}(k)$ is more than the minimum required transfer time $\Upsilon_{k}$, then $z({u_{f}^{k},v}_{f^{\prime }}^{k}\backslash )$must be 1 according to the condition that constraint (18) is satisfied. Otherwise, it must be 0 whether or not constraint (18) is satisfied.

(iv) Capacity constraint on getting-off arcs


$$\sum_{r,s}\sum_{h\in H_{r}}\rho_{r,s}^{h}\left(u_{f}^{k},n_{k}\right)\leq y_{f}(k)\times {Cap}_{f},\forall f\in F;\forall k\in S_{f}\backslash \left\{o_{f}\right\}$$ (20)

Constraint (20) ensures that passengers can get off train f at station k only when this train stops there, namely, $y_{f}(k)=1$; otherwise, they are unable to get off. Hence, the number of passengers visiting getting-off arc $\left(u_{f}^{k},n_{k}\right)$ must be 0 when $y_{f}(k)=0$, while it has no limit when $y_{f}(k)=1$.

Now, a primordial model has been constructed in Section 3, and it is easy to see that all the constraints are linear. However, Eq (1) includes a quadratic term in the calculation of the passenger time cost, namely Eq (3). At the same time, the decision variables of the model include continuous variables and integer variables (binary variables). Therefore, the primordial model is a mixed-integer non-concave and non-linear programming model with the above objective function and constraints. If we want to solve the non-concave and non-linear model with a commercial solver, we can convert the model to a MILP model. It should be emphasized that the primordial model refers to the mixed-integer non-concave and non-linear programming model in Section 3.

## 4 Solution method

In this section, an extended time-dimension method is proposed to convert the mixed-integer non-concave and non-linear programming model to a MILP model. Moreover, a set of simplification strategies is designed to reduce the computational complexity of the MILP method.

### 4.1 Extension time-dimension method

The non-linear part of Eq (1) in the primordial model is the calculation of the total passenger travel time, namely Eq (3), so we need to design a linear method to recalculate it. However, this reformulation will add new decision variables and constraints.

**Fig 4:**
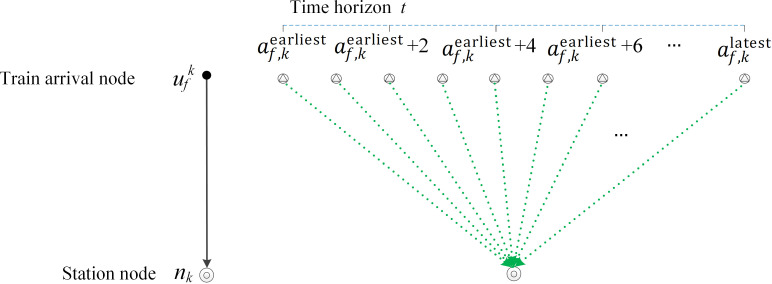
Extension of a getting-off arc considering time dimension.

In order to calculate the total passenger travel time by a linear method, we first need to extend each getting-off arc by considering a time dimension. Specifically, for each getting-off arc $\left(u_{f}^{k},n_{k}\right)$, its arrival node $u_{f}^{k}$ is extended to lots of extended arrival nodes by considering a time dimension, and each extended arrival node corresponds to a possible arrival time $t\in \left[a_{f,k}^{earliest},a_{f,k}^{latest}\right]$ of train f at station k. In Fig 4, $a_{f,k}^{earliest}$ is the earliest arrival time of candidate train f at station k when it departs from the origin station at the earliest departure time $t_{f}^{s}$ and does not stop at any visiting stations, and $a_{f,k}^{latest}$ is the latest arrival time at station k when it departs from the origin station at the latest departure time $t_{f}^{e}$ and stops at any visiting stations. We call this method an extended time-dimension method.

In addition, two new symbols are defined to describe the arrival extended-nodes and getting-off extended-arc in Table 5. According to the extended time dimension and the division accuracy of the extended time dimension, each arrival node can be converted into multiple arrival extended-nodes to replace the role of the arrival node in the space‒time network. Then, as shown in Fig 4, the directed arc constructed from each arrival extended-node to the station node is the getting-off extended-arc. Accordingly, two new auxiliary variables for each getting-off extended-arc are described in Table 6.

**Table 5:** Definitions of arrival extended-nodes and getting-off extended-arc.

Symbols	Descriptions
$u_{f}^{k}(t)$	The arrival extended-node corresponding to arrival time t of arrival node $u_{f}^{k}$
$\left(u_{f}^{k}(t){,n}_{k}\right)$	The getting-off extended-arc from arrival extended-node $u_{f}^{k}(t)$ to station node $n_{k}$

**Table 6:** Definition of two new auxiliary variables on getting-off extended-arc.

Auxiliary variables	Definitions
$\theta \left(u_{f}^{k}(t),n_{k}\right)$	0-1 decision variable, if trainfarrives at stationkat the corresponding timetof getting-off extended-arc $\left(u_{f}^{k}(t),n_{k}\right)$, then $\theta \left(u_{f}^{k}(t),n_{k}\right)=1$; otherwise, it is 0
$\delta_{h}\left(u_{f}^{k}(t),n_{k}\right)$	The number of passengers in time period h finally arriving at destination stationk through getting-off extended-arc $\left(u_{f}^{k}(t),n_{k}\right)$

### 4.2 MILP reformulation

Based on the above new decision variables in Table 6, the calculation of the total travel cost of passengers, namely, Eq (3), can be reformulated as follows:


$$\Psi =\sum_{f}\sum_{r}\sum_{k\in S_{f}\backslash \{{\text{o}}_{\text{f}}\}}\sum_{h\in H_{r}}\sum_{t=a_{f,k}^{earliest}}^{a_{f,k}^{latest}}\delta_{h}\left(u_{f}^{k}(t),n_{k}\right)\times \left(t-R_{h}\right)$$

$$+\sum r\sum s\neq r\sum h\in H_{r}\sum \left(v_{f}^{k},u_{f}^{k^{\prime }}\right)\in A_{T}\rho_{r,s}^{h}\left(v_{f}^{k},u_{f}^{k^{\prime }}\right)\times (l_{k,k^{\prime }}\times \mu_{f})/\sigma$$ (21)

In order to make the new decision variables consistent with the decision variables given in Table 4, some new constraints should be added to the primordial model.

#### (1) Extended Group I: constraints of selection of getting-off extended-arc.

All getting-off extended-arcs are extended from a getting-off arc $\left(u_{f}^{k},n_{k}\right)$, but obviously only one can be selected, namely $\sum_{t=a_{f,k}^{earliest}}^{a_{f,k}^{latest}}\theta \left(u_{f}^{k}(t),n_{k}\right)=1$, when train f is selected to operate, that is $x_{f}=1$. Moreover, train f must arrive at station k at the corresponding time t of getting-off extended-arc $\left(u_{f}^{k}(t),n_{k}\right),$whose value of auxiliary variable $\theta (u_{f}^{k}(t),n_{k}\backslash )$is 1. Hence, the following two constraints must be satisfied.


$$\sum_{t=a_{f,k}^{earliest}}^{a_{f,k}^{latest}}\theta \left(u_{f}^{k}(t),n_{k}\right)=x_{f},\forall \left(u_{f}^{k},n_{k}\right)\in A_{O}$$ (22)

$$\sum_{t=a_{f,k}^{earliest}}^{a_{f,k}^{latest}}\theta \left(u_{f}^{k}(t),n_{k}\right)\times t=a_{f}(k),\forall \left(u_{f}^{k},n_{k}\right)\in A_{O}$$ (23)

Constraint (22) ensures that there is only one getting-off extended-arc with $\theta \left(u_{f}^{k}(t),n_{k}\right)=1$ when train f is selected to run. And constraint (23) further ensures that the arrival time of train f at station k is just the corresponding time t of this getting-off extended-arc.

#### (2) Extended Group II: constraints of passengers visiting getting-off extended-arc.

If passenger wants to get of a train, it is only allowed to choose the getting-off extended-arc of $\theta \left(u_{f}^{k}(t),n_{k}\right)=1$ for getting off train f at station k. Specifically, no passengers choose a getting-off extended-arc of $\theta \left(u_{f}^{k}(t),n_{k}\right)=0$, and all passengers of getting-off arc $\left(u_{f}^{k},n_{k}\right)$ must be distributed on the getting-off extended-arc of $\theta \left(u_{f}^{k}(t),n_{k}\right)=1$. Hence, auxiliary variable $\delta_{h}\left(u_{f}^{k}(t),n_{k}\right)$ satisfies the following two constraints.


$$0\leq \delta_{h}\left(u_{f}^{k}(t),n_{k}\right)\leq \theta \left(u_{f}^{k}(t),n_{k}\right)\times M,$$

$$\left(u_{f}^{k},n_{k}\right)\in A_{O};t\in \left[a_{f,k}^{earliest},a_{f,k}^{latest}\right],\forall h$$ (24)

$$\begin{array}{c} \sum {\mathrm{\backslash nolimits}}_{t=a_{f,k}^{earliest}}^{a_{f,k}^{latest}}\delta_{h}\;\;\left(u_{f}^{k}(t),n_{k}\right)=\sum_{r}\rho_{r,k}^{h}\;\left(u_{f}^{k},n_{k}\right),\forall \left(u_{f}^{k},n_{k}\right)\in A_{O},\forall h \end{array}$$ (25)

Constraint (24) ensures that passengers on the extended getting-off arc must be 0 when $\begin{array}{c} \theta \;\left(u_{f}^{k}(t),n_{k}\right)=0 \end{array}$; otherwise, it can be either 0 or more than 0. M is a large positive number. Constraint (25) further ensures that the number of passengers on all extended getting-off arcs must be the same as on the getting-off arc. Actually, all passengers on the getting-off arc are finally distributed on the extended getting-off arc $\left(u_{f}^{k}(t),n_{k}\right)$, because other extended getting-off arcs are not allowed to have passengers on.

Based on the linear representation of the total travel cost of passengers by Eq (21), the primordial non-concave and non-linear model is converted to a MILP model by making the following two adjustments.

(i) Use Eq (21) to calculate the total travel cost of passengers in Eq (1), that is, replace Eq (3) with Eq (21).
(ii) Add constraints (22)-(25) into the primordial model for making the new variables given in Table 6 consistent with the original decision variables in Table 4.

### 4.3 Simplification strategy

A set of effective simplification strategies is designed to improve the solving efficiency of the MILP model. The passenger flow decision $\rho_{r,s}^{h}(i,j)$ in the model is to decide the number of passengers from the origin station r to destination station s in each time period h visiting each arc in a passenger travel network. Obviously, the total number of passenger flow decisions is the product of the number of passenger flow decisions and arcs, which will be huge. Moreover, the number of constraints related to the decisions, especially the flow balance constraints, is also correspondingly numerous.

Upon examining the passenger flow decisions within the objective and constraints in the MILP model, we identify the following four key characteristics regarding the calculation of passenger numbers:

(1) Eq (21) is only concerned with it in each time period on each extended getting-off arc and when traveling with each travel arc.
(2) Constraint (12) is only concerned with it in each time period on each boarding arc.
(3) Constraints (14)-(17) and (20) are only concerned with it when traveling with these five types of arcs.
(4) Constraint (25) is only concerned with it in each time period on each extended getting-off arc.

In other words, the first half of Eq (21) and constraint (25) are not concerned with the origin stations of passengers; constraint (12) is not concerned with the origin stations and destination stations of passengers; and the second half of Eq (21) and constraints (14)-(17) and (20) are not concerned with the origin stations, destination stations, and time periods of passengers.

Given these observations, it is unnecessary to determine the number of passengers on each arc in detail. Furthermore, none of the passenger flow decisions depend on the origin stations of passengers. Therefore, if we can introduce new variables to refine the passenger flow decisions on the five types of arcs, then it will greatly reduce the number of passenger flow decisions and the amount of calculation of the related constraints. For achieving this aim, Table 7 defines three new decision variables to replace the old decision variables given in Table 4.

**Table 7:** Three new decision variables to describe the number of passengers on the five types of arcs.

Variables	Target Arc	Definitions
$g_{s}^{h}\left(n_{r},j\right)$	Boarding arc	The number of passengers departing from any station to destination station s in time period h on the boarding arc $\left(n_{r},j\right)$
$g_{s}^{h}(i,j)$	Travel arc, dwell arc, and transfer arc	The number of passengers departing from any station to destination station s in time period h on the travel arc, dwell arc, and transfer arc (i,j)
$g_{s}^{h}\left(i,n_{s}\right)$	Getting-off arc	The number of passengers departing from any station to destination station s in time period h on the getting-off arc $\left(i,n_{s}\right)$

Along with this thought, we can use the first new decision variable $g_{s}^{h}\left(n_{r},j\right)$ for the boarding arc to reformulate constraints (12) and (14), the second variable $g_{s}^{h}(i,j)$ for the travel arc, dwell arc, and transfer arc to reformulate both constraints (15)-(17) and Eq (21), and the third variable $g_{s}^{h}\left(i,n_{s}\right)$ for the getting-off arc to reformulate constraints (20) and (25). Actually, these reformulations cannot reduce the number of constraints, but they can efficiently reduce the number of calculations of the related constraints, especially for the flow balance constraints as follows:


$$\sum_{\left(n_{r},j\right)\in {\hat{A}}_{n_{r}}}g_{s}^{h}\left(n_{r},j\right)=\sum_{s\in S\backslash \{r\}}q_{r,s}^{h},\forall h\in H_{r}$$ (26)

$$\sum_{(i,j)\in {\overset{ˇ}{A}}_{j}}g_{s}^{h}(i,j)=\sum_{\left(j,i^{\prime }\right)\in {\hat{A}}_{j}}g_{s}^{h}\left(j,i^{\prime }\right),\forall h\in H_{r};j\in N\backslash \left\{n_{k}:k\in S\right\}$$ (27)

$$\sum_{\left(i,n_{s}\right)\in {\overset{ˇ}{A}}_{n_{s}}}g_{s}^{h}\left(i,n_{s}\right)=\sum_{s\in S\backslash \{s\}}q_{r,s}^{h},\forall h\in H_{r}$$ (28)

Because not all passengers need to visit any station nodes besides those corresponding to their origin and destination stations respectively in the travel process, passengers departing from the station node must therefore be the total demands from the corresponding station, and the number arriving at the station node also should be the total demands arriving at the corresponding station. Thus, constraint (26) ensures that all passengers originally departing from any origin station to their destination station s in time period h must leave the station node $n_{r}$ with the boarding arcs corresponding to that station, namely, the total number of passengers from any stations to their destination stations s in time periodh on all boarding arcs from the station node $n_{r}$ is just the total demands from that station to the destination stations s in the time periodh. Constraint (28) ensures that passengers arriving at the station node $n_{s}$ in the time period h from all connected getting-off arcs must be the total demand to the station s in the time period h no matter from which station. Additionally, constraint (27) ensures that passengers entering an arrival node of station or departure node of station should be equal to that leaving an arrival node or departure node. Finally, we replace the constraints (9)-(11) with constraints (26)-(28).

Next, let us estimate the decrement of constraints caused by the simplification strategy. We denote as | O | , | D | , and | H | the numbers of the origin stations, destination stations, and time periods of passengers respectively; and as | N | the numbers of the nodes of a passenger travel network. The original computation of passenger flow decisions for Eq (21) and the constraints (9)-(11), (12), (14)(11,12,14)-(17), (20)(17,20), and (25) is | O | · | D | · | H | · | N | , and the new computation of passenger flow decisions for Eq (21) and the constraints (12), (14)(12,14)-(17), (20), (25)(17,20,25), and (26)-(28) is | D | · | H | · | N | . Thus, the decrement is | O | · | D | · | H | · | N | - | D | · | H | · | N | . Obviously, this decrement is very big, which can effectively improve the solving efficiency.

Now, an updated new MILP model has been constructed, which consists of Eq (1), (2), and (21); constraints (4)-(8), (12)(8,12)-(20), and (22)-(28). It can theoretically be solved exactly by a commercial solver. However, the specific solving time and efficiency of the solver are closely related to the scale of the input data. In Section 5, we will use a case study of a HSR line in China to verify the feasibility and efficiency of the MILP model.

## 5 Case study

In this section, we apply the MILP model to a case study based on a HSR line in China. Firstly, we describe the Beijing‒Shanghai HSR line and the time-dependent demands for a whole day. Secondly, we use this data as input to the model and obtain a line plan for the day. Thirdly, we construct 17 different cases by varying the number of time periods and candidate trains, and use these 17 cases to explore the solving efficiency of the model. Finally, sensitivity analysis was performed on some parameters of the model. The optimization method is implemented using MATLAB R2019b, GUROBI optimizer version 9.1.1, and the YALMIP toolbox [29]. The model is solved in a workstation equipped with Intel Xeon W-2145 3.70 GHz CPU and 128GB RAM.

### 5.1 Data preparation

**Fig 5:**
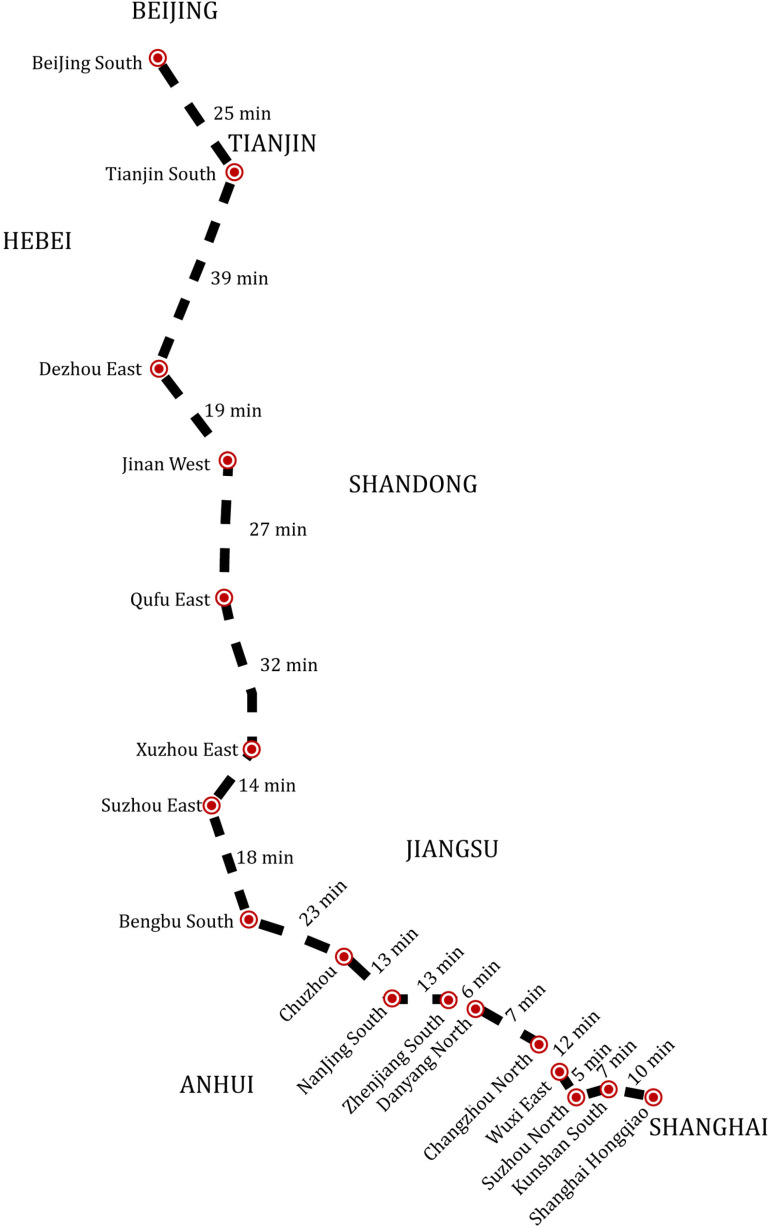
Railway corridor of Beijing–Shanghai HSR (showing 17 of the stations).

We select stations based on total passenger demand data for the entire day from the Beijing‒Shanghai HSR line. As shown in Fig 5, 17 stations are selected from the 23 stations along the line, based on the criterion that each station has more than 1,900 passengers departing in a day (with the median passenger count being 8,725). This results in a railway network consisting of 17 stations and 16 sections, with a total length of 1,318 kilometers, connecting major cities. The study focuses on the single-direction rail route from Beijing South to Shanghai Hongqiao, and since the train capacity is sufficient, we exclude the transfer process. For the candidate trains, 196 trains are pre-determined. The travel times between adjacent stations are computed based on the mileage, assuming a train speed of 350 kilometers per hour. Fig 5 also includes the dwell time $w_{f}^{k}$ for each candidate train f, which are set to 5 minutes, accounting for 2 minutes of stop time, 2 minutes of acceleration time, and 1 minute of deceleration time. The passenger service capacity ${Cap}_{f}$ of each candidate train f is set to 1100 seats/ train-set.

**Fig 6:**
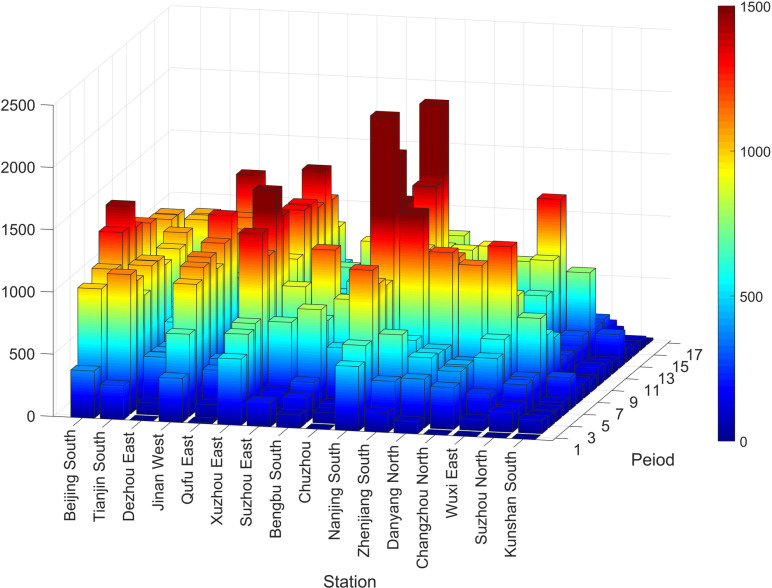
Time-dependent demand of each station in each time period.

Regarding demand data, the operating hours of the stations are from 6:00–23:00, and the day is divided into 17 hourly time periods (e.g., 6:00 ‒ 6:59). The input data includes 136 origin-destination (O-D) pairs, corresponding to a total of 152,937 passengers. The number of passengers departing from each origin station to all destination stations in each time period is shown in Fig 6. Due to the limitation of the 3D figure elements, we do not show the origin station to destination station for each O-D pair in each time period in the whole day. In Fig 6, the large stations have a large number of departing passengers in most of the time periods in the whole day. It can be seen intuitively that there are time ranges in which the number of departing passengers is relatively large, that is from 10:00 to 11:59 and from 17:00 to 18:59, which we artificially call the peak hours.

### 5.2 Line plan

**Fig 7:**
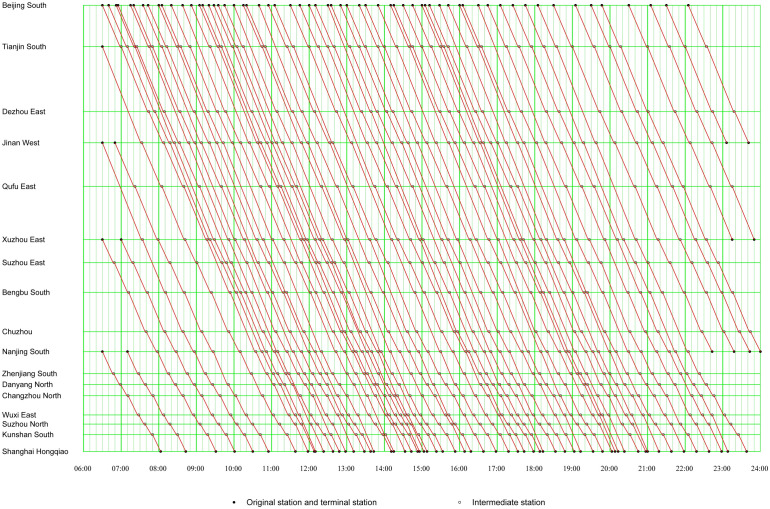
Train diagram.

In general, a train diagram is the materialization of a line plan. It specifies the space‒time distribution of each train and the characteristics of the transit allocation distribution. We used the input data and set the weight parameter ω as 0.5, and the parameter calibration of ω will be introduced later. The outputs of the GUROBI optimizer are listed in Table 8, which are the objective value (Obj val), the operating cost of trains (H), the travel cost of passengers (Ψ), the number of operated trains (#Train), the average waiting time of passengers (Ave time), the average load factor of trains (Ave LF), the CPU time, the optimization gap (Opt gap), the number of constraints (#Cons), the number of continuous variables (#Con), and the number of integer (binary) variables (#Int). The train diagram of the whole day is shown in Fig 7, which shows the origin station, destination station, and intermediate stop stations of each operated train, and their corresponding arrival time and departure time.

**Table 8:** Computational results of the MILP model.

Obj val (10^8^)	H (10^7^)	Ψ (10^8^)	#Train	Ave time	Ave LF	CPU time	Opt gap	#Cons	#Con	#Int
2.6163	2.8594	4.9467	68	6.0433 m	62.89%	279,996 s	5.5%	2,213,880	1,900,632	66,979

#### (1) Space–time distribution of train routes.

**Fig 8:**
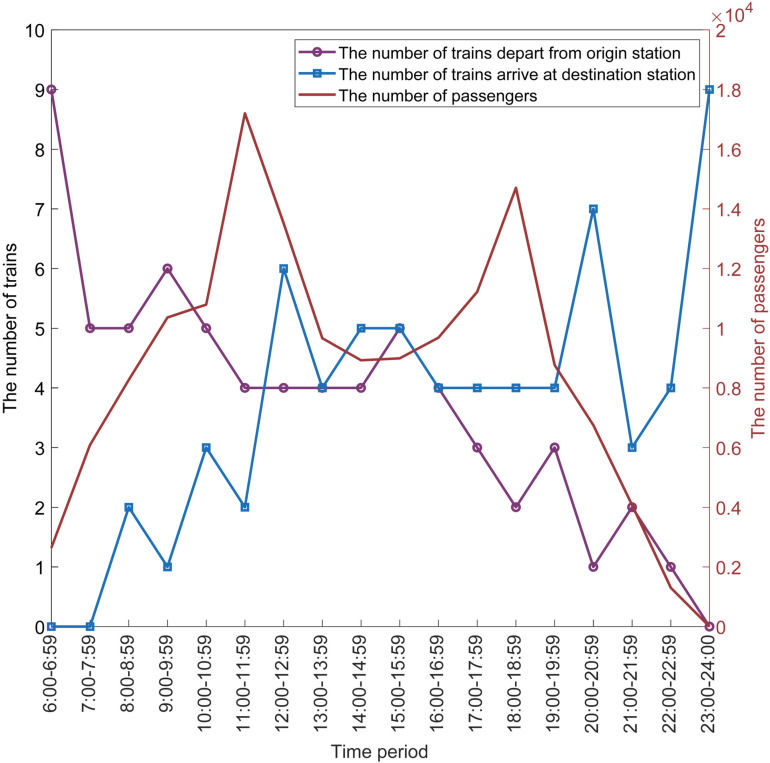
Variation of the number of trains departing from origin stations, the number of trains arriving at destination stations, and the number of passengers in each time period.

There are 53 trains with an operation mileage of 1,318 km and 15 trains with less than 1,318 km, accounting for 77.9% and 22.1% of the total operated trains, respectively. Most of the trains depart from Beijing South, including 61 trains from Beijing South, 1 from Tianjin South, 2 from Jinan West, 2 from Xuzhou East, and 2 from Nanjing South. In addition, most of the trains arrived at Shanghai Hongqiao, including 60 trains at Shanghai Hongqiao, 4 at Nanjing South, 2 at Xuzhou East, and 2 at Jinan West. Fig 8 illustrates the variation of the number of trains that departed from the origin station, arrived at the destination station, and for each different time period. There are 4 time periods, 6:00 to 6:59, 9:00 to 9:59, 15:00 to 15:59, and 19:00 to 19:59, that have the most operated trains. The peak hours of the input data are 10:00 to 11:59 and 17:00 to 18:59 in Fig 5, and the time periods of 9:00 to 9:59 and 15:00 to 15:59 which have most trains in Fig 7 are 1–2 hours earlier than the peak hours of input data. The other two time periods of 6:00 to 6:59 and 19:00 to 19:59 which have the most trains are in the morning and evening. This shows that the line plan generated by the MILP method can combine the TOC and PTC well in the directed graph. This can not only enable the line plan to better service the passengers but also make the passengers choose a suitable time to take their trains.

**Fig 9:**
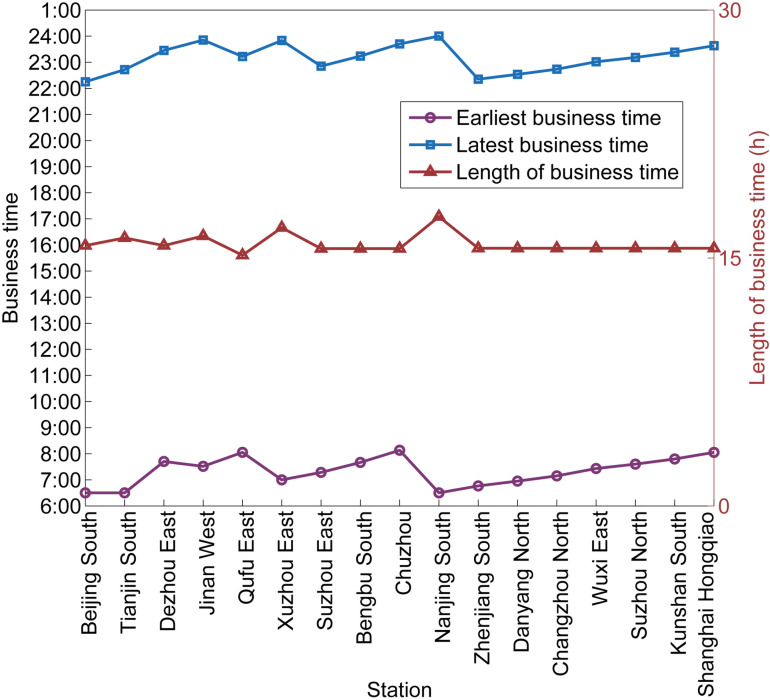
Variation of the business times of each station.

The train routes are evenly distributed in Fig 7, and the train routes are relatively closely distributed during the peak hours, while the train routes are relatively loosely distributed during the non-peak hours. All operated trains leave from the origin stations from 6:30 to 22:10 and arrive at their destination stations from 8:00 to 24:00. Because we set the middle time ${ℛ}_{h}$ of the first time period (6:30) as the time when the time-dependent demands enter their stations, the results show that the earliest departure time of the trains in the morning is later than 6:30. Fig 9 illustrates the variation of the earliest business time, the latest business time, and the length of business time of each station. The earliest departure time at Shanghai Hongqiao is relatively late in the morning and the latest departure time at Beijing South is relatively late in the evening, because we are only considering a single direction. In addition, Tianjin South, Jinan West, Xuzhou East, and Nanjing South all have the relatively earliest and latest business times and the length of their business times is more than 16 hours, so passengers can have more times to choose trains with longer business times. This shows that the line plan with time information can possibly reflect the business times of stations which are influenced by the time-dependent demand. Another level also shows that time-dependent demand can more accurately influence the line plan generated by the MILP method.

#### (2) Stop frequency at station.

**Fig 10:**
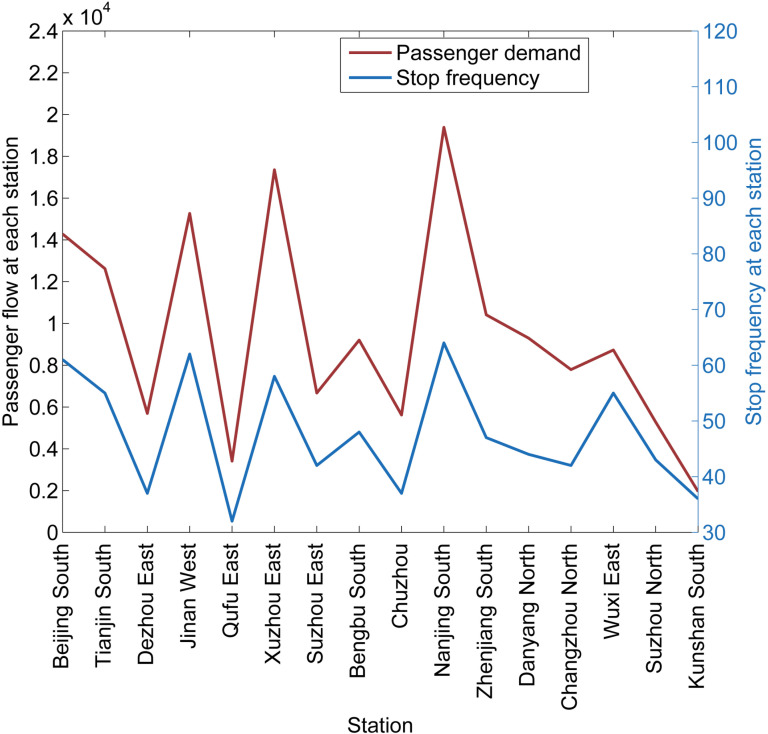
The passenger demand and stop frequency at each station.

In Fig 10, the red and blue lines represent passenger demand and stop frequency, respectively, aiming to illustrate the correlation between the two. Passengers departing from each station fluctuated from 1953 to 19,385, and the stop frequency at each station fluctuated from 32 to 64. Intuitively, the variation of the two curves is very consistent, which indicates that the passenger demand and train stop frequency at each station match very well. The MILP model enables the passengers to find appropriate PTC on the directed graph. This shows that the stop schedule in the line plan generated by the MILP model can meet the time-dependent demand well, and matching the stop schedule with the time-dependent demand can not only provide reasonable stop schedules for the line plan but also provide reasonable trains for passengers.

**Fig 11:**
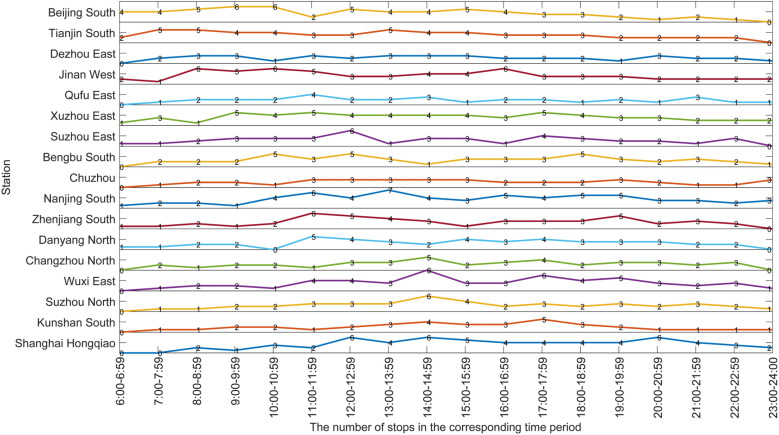
The stop frequency at each station in each time period.

Next, Fig 11 shows the stop frequency at each station in each time period. We can intuitively see the variation of the number of stops at each station in all time periods of the whole day. The maximum number of stops per station in each time period is generally in the time ranges of 10:00 to 14:00 and 17:00 to 20:00, which coincides with the peak hours of the time-dependent demand in Fig 6. This shows that the line plan meets the time-dependent demand very well from a more microscopic perspective. The five stations with the ability to serve as origin stations have 2–7 trains stopping at their stations in most of the time periods, the other stations have 1–6 trains stopping at their stations in most of the time periods, and some stations have no trains stopping at them for only a few periods. This shows that larger stations with the ability to serve as origin stations have more stops in most of the time periods and passengers departing from these stations have better train choices.

#### (3) Average waiting time of passengers and average load factor of trains.

Table 9 and Table 10 summarize the Ave time and Ave LF. The proportion of operated trains with an Ave time of less than 10 minutes is 86.29%, and less than 5 minutes is 56.87%. This shows that the line plan can allow most passengers to have a relatively short waiting time and the MILP method reduces the travel cost of passengers well. As for the Ave LF, those whose value is higher than 55% account for 54.41%, those whose value is lower than 35% account for 26.47%, and those whose value is higher than 35% and lower than 55% account for 19.12%. This shows that the line plan can allow the railway company to use more trains with a higher load factor. However, 26.47% of trains have a relatively low Ave LF, because all the time-dependent demand must take trains to their destinations, and the method will therefore provide trains that have only a few passengers.

**Table 9:** Distribution of Ave time for all operated trains.

Ave time (m)	[0, 5]	(5, 10]	(10, 20]	Above 20
# Train	38	20	9	1
Proportion of 68	56.87%	29.42%	12.24%	1.47%

**Table 10:** Distribution of Ave LF for all operated trains.

Ave LF (%)	[0, 35]	(35, 55]	(55, 75)	(75, 90)	Above 90
# Train	18	13	9	4	24
Proportion	26.47%	19.12%	13.24%	5.88%	35.29%

**Fig 12:**
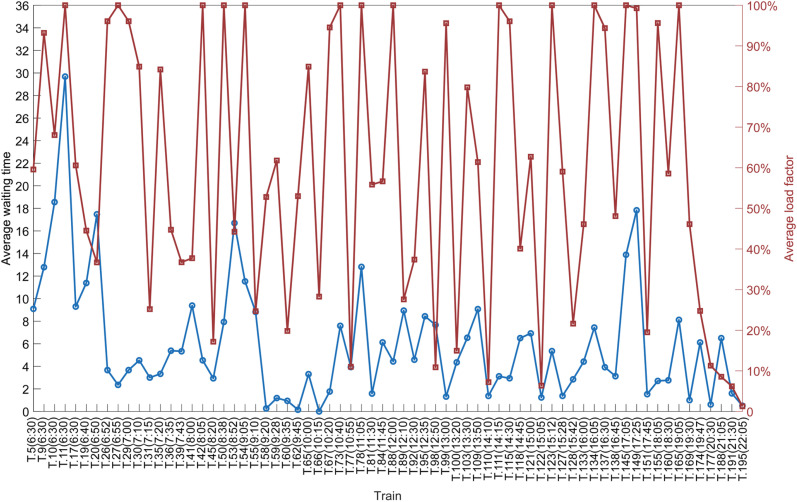
The correlation between the Ave time and Ave LF of each train.

**Fig 13:**
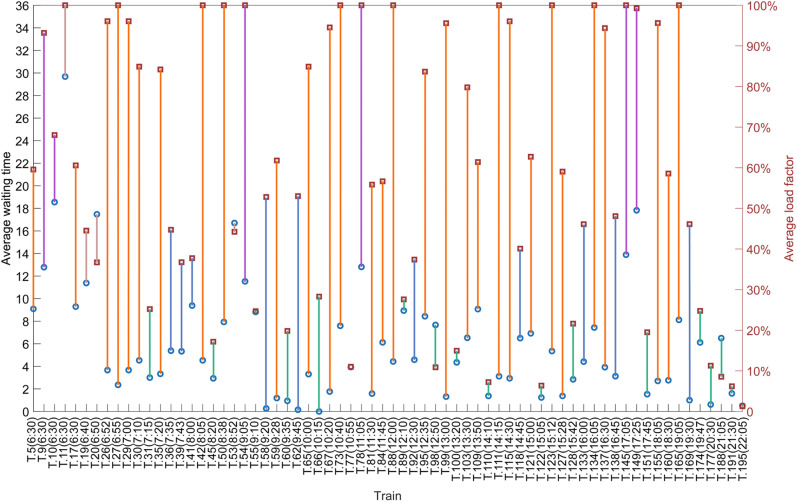
Auxiliary figure: the correlation between the average waiting time and average load factor of each train.

Next, we draw the correlation between the Ave time and Ave LF of each train in Fig 12, and the abscissa represents the operated train from the candidate trains (for example, T.5(6:30) represents the 5^th^ candidate train and this train departs from its origin station at 6:30). Fig 13 is used to give an auxiliary description of the correlation between the Ave time and Ave LF. If we consider that an Ave LF of trains greater than 55% and an Ave time of passengers less than 10 minutes are relatively good results, then we can summarize this fluctuating trend shown in Fig 13 with Table 11.

**Table 11:** Distribution of average waiting time and average load factor.

Distribution range	# Train	Proportion	Line color in Fig 13
Ave time < 10m & Ave LF > 55%	30	44.12%	yellow
Ave time < 10m & Ave LF < 35%	18	26.47%	green
Ave time < 10m & 35% < Ave LF < 55%	10	14.71%	blue
10m < Ave time < 20m & Ave LF > 55%	6	8.82%	purple
Ave time > 10m & Ave LF < 35%	0	0%	no

The proportion of the operated trains with an Ave time of less than 10 minutes and an Ave LF greater than 55% is 44.12%, and these trains are evenly distributed (yellow line in Fig 13) in all time periods of the whole day. We consider that these trains have high quality in the line plan, because they have both a high Ave LF for the railway company and a low Ave time for passengers. For example, T.111 departs from Beijing South at 14:15 and finally arrives at Shanghai Hongqiao at 19:48, it passes 12 stations including Tianjin South, Jinan West, Xuzhou East, and Nanjing South, and the Ave time and the Ave LF are 3.12 minutes and 100% respectively. The proportion of the operated trains with an Ave time greater than 10 minutes and an Ave LF of less than 35% is 0%, indicating that the line plan has no operated trains with very poor quality.

In addition, the proportion of operated trains with an Ave time of less than 10 minutes and an Ave LF of less than 35% is 26.47% (green line in Fig 13), which we consider to be of low quality in the line plan. There are 13 trains loosely distributed in the time periods from morning to afternoon, and the other 5 trains are intensively distributed after 19:30. For example, T.45 departs from Beijing South at 8:20 and finally arrives at Shanghai Hongqiao at 13:43 passing 10 stations, and the Ave time and the Ave LF are 2.94 minutes and 17.17% respectively. T.42 in front of T.45 in Fig 7 departs from Beijing South at 8:05 and finally arrives at Shanghai Hongqiao at 13:38, and its Ave time and Ave LF are 4.53 minutes and 100% respectively. However, these two operated trains have nearly identical running times and stop schedules. T.42 carried most of the passengers from the neighboring time periods, but there were a few passengers who needed to travel in those neighboring time periods, so T.45 was operated so that all the passengers in those neighboring time periods could get on a train to their destination. In Fig 13, we can see that almost all of the green lines have a yellow line in front of them, except for the 5 trains after 19:30, which shows that this group of trains is similar to the relationship between T.42 and T.45. In addition, we can see that the 5 trains after 19:30 are all green lines, because the time-dependent demand is small at night, but they must be operated so that all passengers can take a train to their destination. This could be because the MILP model sets all trains to the same capacity. If different passenger service capacities could be set, this issue could be resolved.

### 5.3 Efficiency analysis

**Fig 14:**
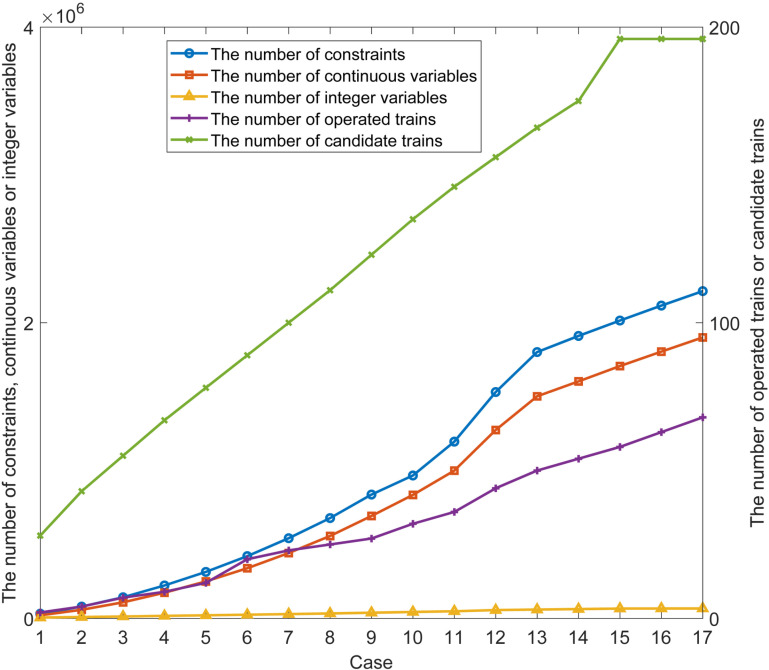
Variation curve of the relevant factors of the input and output data.

**Fig 15:**
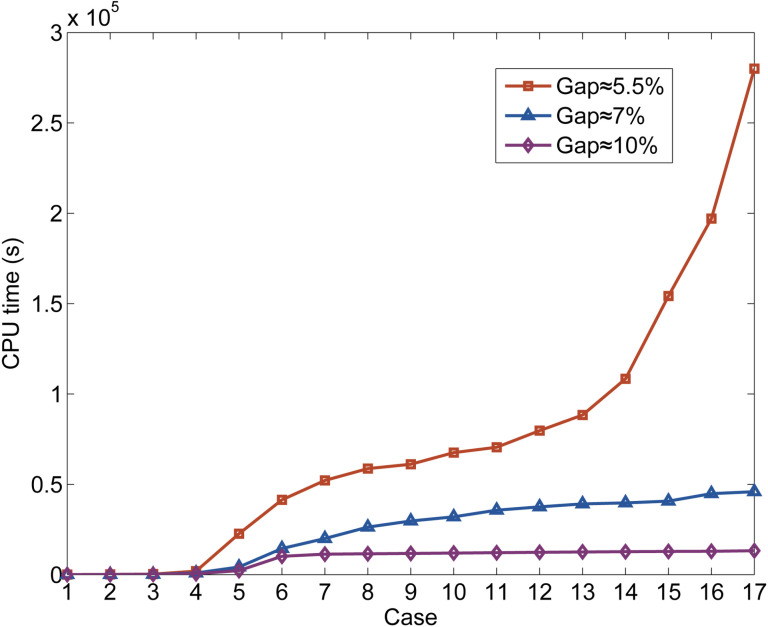
Variation curve of the CPU time with different gaps.

In order to further analyze the solving efficiency of the MILP model, we constructed 17 cases of different data scales based on the input data. The construction of these 17 cases is based on selecting different parts of the time-dependent demands and candidate trains in the whole day. We want to summarize and analyze the calculation results of these 17 cases, so as to roughly summarize the solving efficiency of the MILP method for different data scales. In Table 12, we list the relevant factors that describe the scale characteristics of these 17 cases, which are respectively the selected time range of time-dependent demands, the number of candidate trains (#Can train), #Cons, #Con, and #Int. Next, we use these 17 cases to repeatedly calculate and obtain multiple sets of results. Table 12 also lists the CPU times of these 17 cases when the optimization gap is about 10%, 7%, and 5.5% respectively, and lists the number of operated trains in the line plans when the optimization gap is about 5.5%. We have drawn Fig 14 and Fig 15 to show more clearly the data listed in Table 12.

**Table 12:** The characterization of the 17 cases.

Case	Time range	#Can train	#Cons	#Con	#Int	CPU time (s) with different gaps			#Train gap (5.5%)
						10%	7%	5.5%	
1	6:00-6:59	28	31,585	26,350	5353	5	5	15	2
2	6:00-7:59	43	79,743	67,320	9201	48	68	117	4
3	6:00-8:59	55	142,693	121,390	12,888	160	186	274	7
4	6:00-9:59	67	222,582	190,503	16,821	500	903	1894	9
5	6:00-10:59	78	314,396	270,551	20,677	2232	4230	22,637	12
6	6:00-11:59	89	420,961	363,939	24,700	10,167	14,444	41,370	20
7	6:00-12:59	100	542,309	470,680	28,888	11,339	19,967	52,135	23
8	6:00-13:59	111	678,506	590,810	33,244	11,513	26,298	58,674	25
9	6:00-14:59	123	837,605	731,057	38,138	11,721	29,666	61,089	27
10	6:00-15:59	135	966,555	878,602	43,166	11,962	32,001	67,533	32
11	6:00-16:59	146	1,195,620	1,047,790	48,142	12,101	35,679	70,501	36
12	6:00-17:59	156	1,531,142	1,330,259	56,065	12,356	37,523	79,666	44
13	6:00-18:59	166	1,801,515	1,561,757	59,621	12,588	39,121	88,332	50
14	6:00-19:59	175	1,910,685	1,666,330	62,988	12,643	39,687	108,346	54
15	6:00-20:59	196	2,014,935	1,773,241	66,331	12,801	40,666	154,153	58
16	6:00-21:59	196	2,115,917	1,871,259	66,736	12,919	44,795	197,034	63
17	6:00-22:59	196	2,213,880	1,967,611	66,979	13,201	45,874	279,996	68

Fig 15 shows the variation curves of the CPU times for these 17 cases under different optimization gaps. The red line (gap ≈ 5.5%) shows a slow increase from the 5th case to the 14th case and an obvious increase from the 15th case. This indicates that when the calculated scale of the case is lower than the scale represented by the 14th case, the CPU time will not increase significantly with the increase of the scale. The input data for the 14th case contains 175 candidate trains, 1,910,685 constraints, 1,666,330 continuous variables, and 62,988 integer variables, etc., such that the scale can represent a medium- to large-scale HSR line. And when the calculated scale of the case is higher than the scale represented by the 15th case, the CPU time will increase significantly with the increase of the scale. The purple (gap ≈ 10%) and blue (gap ≈ 7%) lines show that the CPU time increases slowly when the optimization gap is set to a relatively loose value.

**Fig 16:**
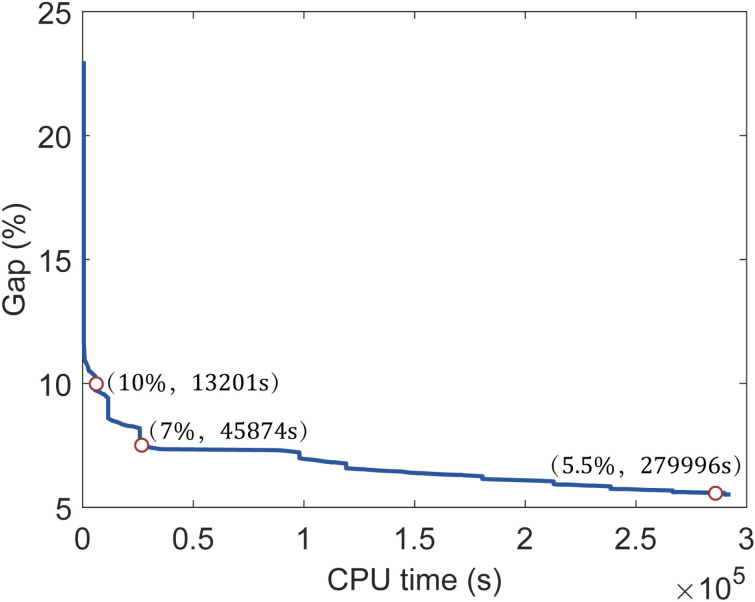
Variation curves of the CPU time with gap for the 17^th^ case.

In addition, we drew Fig 16 to show the variation curves of the CPU time with the gap for the 17th case. As can be seen from the figure, it took 13,201 seconds for the gap to drop to 10%, 32,673 seconds for the Gap to drop from 10% to 7%, and 234,122 seconds for the gap to drop from 7% to 5.5%. This indicates that the value of the objective converges rapidly in the early stage of the solution and converges slowly in the later stage. When the decision-maker can accept a relatively high convergent gap, this MILP method can find a satisfactory solution very quickly, especially for large-scale problems, which can be solved very efficiently. However, when the decision-maker needs a very low convergent gap, the CPU time for large-scale problems will be greatly increased.

### 5.4 Sensitivity analysis

The setting of coefficient ω and the parameter of the MILP model will affect the solution result of the model. We want to analyze the variation of these values affecting through sensitivity analysis. From the input data, we select four time periods from 6:00 to 9:00 departing from each station exceeding 1900 passengers, thus generating a case consisting of 10 stations, 9 sections, and 43 O-D pairs.

#### (1) Impact analysis of ω.

The value of ω can affect the ratio of two parts of the objective. We want to observe the variation of the value of the objective by changing the value of ω to achieve the purpose of calibrating the coefficient.

**Fig 17:**
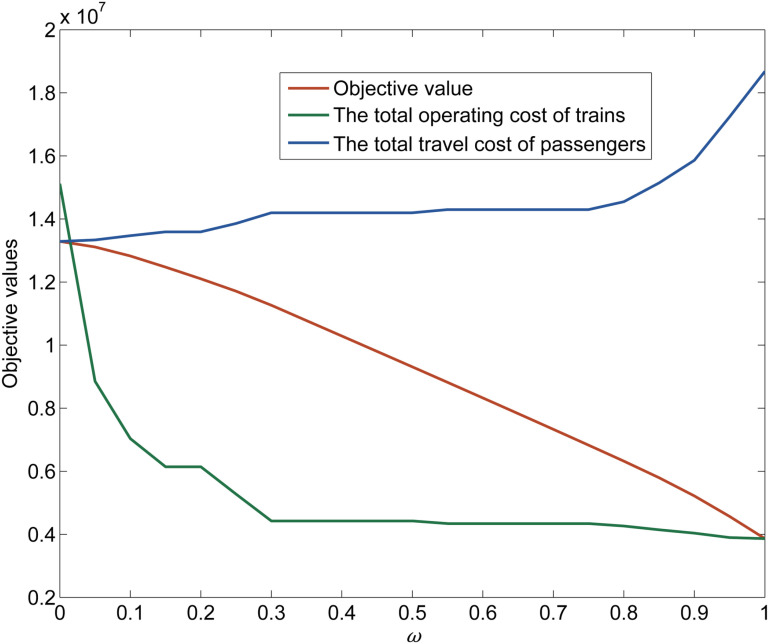
Variation of the objective with ω∈[0, 1].

**Fig 18:**
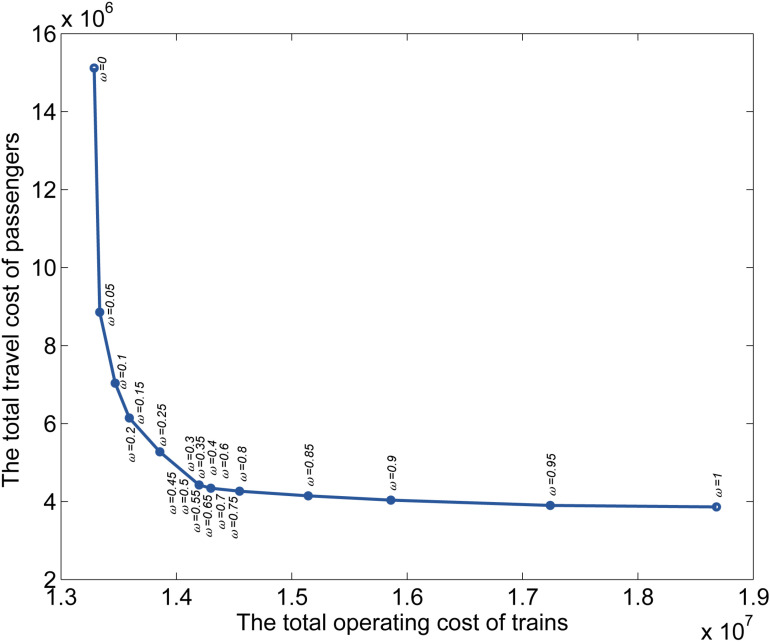
Variation between the 𝐇 and Ψ with ω∈[0, 1].

Fig 17 shows the relationship of the objective when ω varies from 0 to 1. The relationship between the operating cost and the travel cost when ω varies from 0 to 1 can be seen more intuitively in Fig 18. The total operating cost varies greatly when ω varies from 0 to 0.3, and the total travel cost varies greatly when ω varies from 0.75 to 1. This indicates that the variation of ω will greatly affect the values of the operating cost and the travel cost in these two ranges respectively. That is, when the value of ω is set from 0 to 0.3, the solution of the MILP model tends to minimize the total operating cost more obviously than when ω is set to other values. And when the value of ω is set from 1 to 0.75, the solution of the MILP model tends to minimize the total travel cost more obviously than when ω is set to other values. This can help decision-makers to set different values of ω more intuitively and quantitatively according to the different goals they want to optimize.

In the two ranges of ω values from 0.3 to 0.5 and from 0.55 to 0.75, the values of the total operating cost are 0.4425 × 10^7^ and 0.4341 × 10^7^, and the values of the total travel cost are 1.4198 × 10^7^ and 1.4299 × 10^7^, respectively. Therefore, the operating cost and travel cost stay pretty much the same when the value of ω is in these two ranges, and the value of the objective decreases like a direct proportion function. It can also be seen from Fig 18 that when ω varies in these ranges, the points related to the operating cost and travel cost are concentrated at two points. When the value of ω is set in these ranges, the values of the operating cost and travel cost remain stable, which can greatly reduce the violent fluctuation and instability of model optimization. Therefore, we suggest setting the value of ω in the range from 0.3 to 0.75.

#### (2) Impact analysis of passenger service capacity.

**Fig 19:**
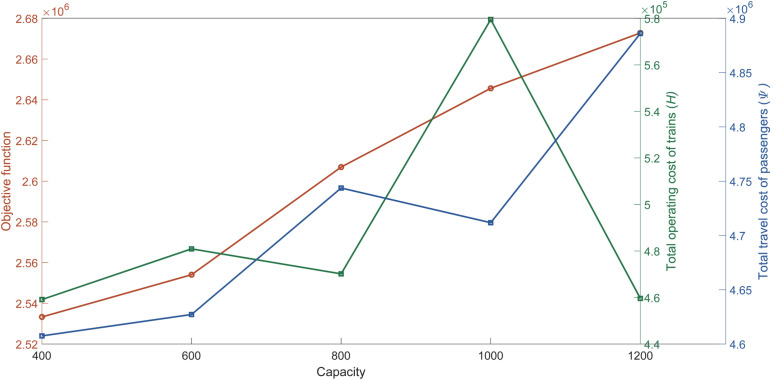
Variation curves of the value of the objective function with different passenger service capacities.

We want to analyze the influence of the capacity (Cap) of trains on the MILP model. We fix the candidate trains and vary the value of Cap from 400 seats/ train-set to 1200 seats/ train-set with a step of 200 seats/ train-set. The organization cost and train travel cost per unit of engine time of each train are reduced with the reduction of Cap. The results are shown in Table 13 and the varying curves of the value of the objective with different Caps is shown in Fig 19.

**Table 13:** Computational results of different passenger service capacities.

Cap	400	600	800	1000	1200
Organization cost	20,000	30,000	40,000	50,000	60,000
Travel cost	80	110	140	170	200
Obj val	2,533,291	2,554,041	2,606,922	2,645,624	2,672,817
H	459,120	480,900	470,160	579,480	459,600
Ψ	4,607,463	4,627,182	4,743,684	4,711,768	4,886,034
#Train	11	8	6	6	4
Ave time	12m 10s	12m 11s	14m 55s	14m 33s	18m 28s
Ave LF	68.26%	62.57%	62.57%	50.06%	62.57%
CPU time	3 m 30s	1m 2s	1m 18s	45s	22s

The values of the objective, the operating cost, the travel cost, the Ave time, and the Ave LF are minimum when the Cap is 400. The values of the objective, the total travel cost, and Ave time are maximum when the Cap is 1200. The value of the objective function increases with the increase of the Cap, and the value of the total travel cost increases with the increase of the Cap (except the decrease when the Cap increases from 800 to 1000). The value of the total operating cost fluctuates with the increase of the Cap, and is almost equal when the Cap is 400 and 1200. The line plan can arrange more small marshalling trains under the same operating cost when the Cap is 400, so it can provide more suitable trains to meet the demands and save more operating costs for railway companies. However, the line plan needs to arrange more big marshalling trains according to the demands, because the trains on the HSR (especially on the China HSR) need to complete the task of transporting a large number of passengers within a certain time range.

In addition, the value of the total travel cost decreases, and the value of the total operating cost increases when the Cap increases from 800 to 1000. The Ave time and Ave LF are smaller when the Cap is 1000, and the number of operated trains in the two generated line plans is the same at six. In other words, when the number of operated trains in the line plan is fixed, the larger the Cap is, the more comfortable the train decisions that will be provided for passengers. However, the Cap cannot be enlarged without limit, because it is constrained by the operating cost. The setting of the Cap is a complex problem, it is affected by the distribution of passengers and the related operating cost of railway companies, etc., and its setting problem cannot be comprehensively demonstrated through this simple case. In this article, the model does not consider the different Caps, and the Cap is set to a fixed value.

## 6 Conclusions

This paper addresses the line planning problem (LPP) that simultaneously optimizes both train and passenger time in a universal passenger railway system with passenger train choice (PTC) under time-dependent demand. Based on a physical infrastructure-based directed graph, the practical problem is formulated as a mixed-integer non-concave and non-linear programming model to optimize both the train operation choice (TOC), considering train arrival/departure times, and PTC, considering passenger time preferences. Then, the non-concave and non-linear model is converted to a mixed-integer linear programming (MILP) model by a designed extended time-dimension method, and a set of effective simplification strategies is proposed to improve the efficiency of the model. It is the first to propose a MILP method to solve the time-dependent demand-driven line planning with diverse goals and obtain a global optimization solution in a passenger railway system.

A case study is used to verify the feasibility and efficiency of the MILP method based on the simplified Beijing‒Shanghai HSR line and a set of time-dependent demands for a whole day. The method not only greatly improves the operational efficiency of the passenger railway system but also accurately meets the diverse travel preferences of time-dependent demand. It has good solution efficiency for medium- to large-scale HSR lines. The generated line plan can balance the total operating cost of trains and the total travel cost of passengers well, thus achieving the goals of matching the time-dependent demand, ensuring the service level, and improving the utilization rate of the transportation capacity.

Future research work can be further extended in some possible aspects. First, it would be interesting to try a decomposition method or the relaxation method to solve the relevant model ‒ methods that are usually used to find an acceptable solution in a reasonable CPU time. Second, further consideration will be given to more practical situations in the model, such as a multi-mode passenger transportation system combining a railway and subway. Third, this optimization problem can further integrate pricing and seat allocation. In this context, it is necessary to consider an elastic demand, and it will be difficult to find a method to convert the integrated model to a linear model. At the same time, it will further increase the complexity of the model formulation and solution method.

## Supporting information

S1 File (RAR)
